# Effects of Seasonal Heat Stress during Late Gestation on Growth Performance, Metabolic and Immuno-Endocrine Parameters of Calves

**DOI:** 10.3390/ani12060716

**Published:** 2022-03-12

**Authors:** Cheng Tang, Yan Liang, Jiahe Guo, Mengqi Wang, Mingxun Li, Huimin Zhang, Abdelaziz Adam Idriss Arbab, Niel A. Karrow, Zhangping Yang, Yongjiang Mao

**Affiliations:** 1 Key Laboratory for Animal Genetics, Breeding, Reproduction and Molecular Design of Jiangsu Province, College of Animal Science and Technology, Yangzhou University, Yangzhou 225009, China; chengtang_0303@163.com (C.T.); mz120181016@yzu.edu.cn (Y.L.); gjh18752783920@163.com (J.G.); mengqi.wang.1@ulaval.ca (M.W.); limingxun@live.com (M.L.); minmin-911@163.com (H.Z.); arbabtor@yahoo.com (A.A.I.A.); yzp@yzu.edu.cn (Z.Y.); 2 Joint International Research Laboratory of Agriculture and Agri-Product Safety, Yangzhou University, Yangzhou 225009, China; 3 Center for Genetic Improvement of Livestock, Department of Animal Biosciences, University of Guelph, Guelph, ON N1G 2W1, Canada; nkarrow@uoguelph.ca

**Keywords:** heat stress, late gestation, hormone, immunity, calves

## Abstract

**Simple Summary:**

Heat stress encountered during late gestation will not only reduce subsequent lactation performance, it will also affect the health and growth performance of calves. This study investigated the effects of seasonal heat stress during late gestation on the growth, metabolism, and immunity of calves. We found that heat stress during this period can impair hormones, oxidative stress, and immunity of calves before weaning and increase the risk of diarrhea in the first week after birth.

**Abstract:**

Heat stress during late gestation could affect subsequent lactation performance, resulting in damage to the immune function, health, and growth performance of calves. This study aimed to compare the effects of 33 days of summer stress (Summer group, 70.15 < THI < 74.28) with 33 days of winter during late gestation (Winter group, 57.55 < THI < 67.25) on the growth, hormones, oxidative stress, and immune function of calves. Calves (Summer, *n* = 28; Winter, *n* = 23) were separated from cows immediately after birth and fed with 2 L colostrum within 2 h and 8–10 h after birth, respectively, and weaned at 60 days of age. Bodyweight (BW) was measured at birth and weaning. Withers height (WH), body length, and chest girth were measured at birth, 30 days, and 60 days of age. The health of calves ranging in age from 1 to 7 days was recorded. Plasma interferon-γ (IFN-γ), superoxide dismutase (SOD), adrenocorticotropin (ACTH), gonadotropin-releasing hormone (GnRH), IgG, cortisol, heat shock protein 70 (Hsp70), growth hormone (GH), insulin, lipid peroxide (LPO), and tumor necrosis factor-α (TNF-α) levels were measured in calves at 0 (before colostrum feeding), 3, 7, 14, 28, and 56 days of age. The pregnancy period of the Summer group was shortened by 1.44 days. The Winter and Summer groups had the same birth weight. One week after birth, the incidence of diarrhea was 57.14% and 21.74% in Summer and Winter groups, respectively. Compared with the Winter group, TNF-α in the Summer group increased significantly before colostrum feeding. ACTH and LPO decreased significantly at 3 days of age, ACTH and TNF-α decreased significantly at 7 days of age, Hsp70 increased significantly, ACTH was significantly reduced at 14 days of age, and Hsp70 increased dramatically at 7 days of age. SOD and TNF-α increased statistically at 28 days of age, LPO decreased significantly, and IFN-γ decreased significantly at 56 days of age, while IgG and GH increased significantly. We conclude that maternal heat stress during late gestation can damage the oxidative stress and immune plasma indexes of offspring before weaning.

## 1. Introduction

Heat stress disrupts the dairy cow’s production cycle, causing decreased feed intake, metabolic shift, and ultimately decreased production efficiency [1]. Furthermore, heat stress during the dry period harms the function of immune cells in dairy cows preparing for calving, and this effect has been extended to the following lactation [2]. Calves that have been subjected to heat stress have shown a reduction in growth both before and after weaning [3]. Moreover, heat stress during late gestation significantly impacts dairy calves’ health, growth, and ultimate performance [4]. Therefore, the problem of heat stress in summer is of great concern as it has a severe impact on the entire production cycle of dairy cattle and has become one of the most serious challenges faced by dairy cattle breeders due to the continuous rise in global temperature [5] and intensification of animal farming around the globe [6].

When dairy cows are exposed to heat stress, they experience increased rectal temperatures and respiratory rates and reduced lying time [7]. Heat stress during lactation also changes metabolic rate and reduces feed intake, both resulting in decreased milk yield [8]. The cooling of heat-stressed dairy cows after delivery is beneficial to control and maintain the metabolic compliance changes needed for the beginning of lactation, while ensuring the proper control of inflammation and oxidative metabolites [9].

Pregnancy and the maternal environment during pregnancy are determinants of adult phenotypes of offspring [10]. Perturbations in the intrauterine environment, from maternal malnutrition and stress to elevated body temperature during pregnancy, can induce structural and functional changes in the fetus which may last into adulthood [11,12,13]. Late gestation is an important period in the dairy cow’s production cycle. In this stage, maternal heat stress during gestation affects the growth and physiology of fetal development [14,15,16]. Late gestation is characterized by increased muscle growth and fat deposition, which contributes to the rapid increase in fetal weight [17]. At this stage, the fetus has reached approximately 60% of its birth weight [18], and the cow’s mammary tissues grow and renew [19] and organs undergo functional maturity to prepare for the maintenance of life outside the womb [20]. Therefore, when cows are subjected to heat stress in this stage, subsequent lactation can be affected, which can impact calf health. Heat stress during late gestation has been shown to reduce calf birth weight, which remained significantly reduced at 7 days of age, according to Trifkovi et al. [21]. Monteiro et al. [22] found that heat stress in the last 6 weeks of pregnancy harmed the ability of calves to acquire passive immunity. In addition, study results from Tao et al. [23] showed that serum IgG concentrations of calves born under heat stress in late gestation were significantly lower than calves born after cooling treatment during the same period. At present, most studies on heat stress in late gestation are based on the model of heat stress without cooling treatment. Currently, there are few studies on the comprehensive effects of seasonal heat stress during late gestation on the growth performance and metabolic and immuno-endocrine functions of preweaned dairy calves.

The objective of this study was to determine whether seasonal heat stress during late gestation would affect calf growth and metabolic and immuno-endocrine functions. We hypothesized that late gestation calves exposed to no heat stress would perform optimally in terms of growth and metabolic and immuno-endocrine functions when compared with late gestation heat stress-exposed calves.

## 2. Material and Methods

### 2.1. Animals and Experimental Design

All experiments were performed according to the care and use guidelines of experimental animals established by the Ministry of Science and Technology of the People’s Republic of China (approval number 2006-398). The sample collection process was in line with the welfare ethics of experimental animals, a production license of experimental animals was obtained (SYDW-2019005), and the experimentation was also approved by Yangzhou University, Yangzhou, China. The experiment was conducted at Yonghao Dairy Farm in Suining, Xuzhou, Jiangsu Province, from 2 August 2018 to 21 January 2019.

Fifty-one Holstein female calves were used for the study. The heat stress season (Summer group) included 28 calves from dams exposed to the summer season during late gestation, and the non-heat stress season (Winter group) included 23 calves from dams who carried out their late gestation during the winter season. All cows during the experiment were kept in barns during their respective periods of late gestation. All cows were fed, twice daily, the same total mixed ration (TMR) (Table 1) at 09:00 and 15:00. TMR push-up was carried out once every half hour to ensure the cows could feed freely and they had free access to drinking water. During the experiment, the feeding management and nutrition levels were the same for each season. The energy, protein, mineral, and vitamin levels in the diet were based on the feeding standards for Chinese dairy cows. Within 2 h of birth and 8–10 h later, calves were fed 2 L colostrum with a Brix level of more than 20%. All calves were then managed by moving them to the calf unit where they were, individually housed in hutches, and weaned at 60 days postpartum. To provide ample drinking water, the water was changed twice a day.

### 2.2. Growth Measures and Sample Collection

The ambient temperature and relative humidity were measured in the barn for the gestating dry cows. Additionally, the rectal temperature of the cows was recorded daily at 10:00 and 14:00. Furthermore, the average records of the temperature–humidity index (THI) and rectal temperature were calculated. The THI was calculated using the Dikmen et al. equation [24]: THI = (1.8 × T + 32) − [(0.55 − 0.0055 × RH) × (1.8 × T − 26) ], where T = air temperature (°C) and RH = relative humidity (%). All calves were weighed by electronic scales at birth and weaning. Withers height (WH), body length, and chest girth of calves were measured at birth and at 30 and 60 days of age.

Blood samples were collected from the jugular vein of all calves into ethylene diamine tetraacetic acid (EDTA) anticoagulant vacutainer tubes at 0 (before feeding colostrum), 3, 7, 14, 28, and 56 days of age. The blood was collected before each feeding, after which it was mixed at room temperature and the plasma was separated at 4 °C by centrifugation for 30 min at 3000× *g*, then stored frozen (−20 °C) until assay time. In this experiment, an individual Enzyme Linked Immunosorbent Assay Kit (ELISA kit, Shanghai Yanjin Biological, Shanghai, China) was used to detect interferon-γ (IFN γ), superoxide dismutase (SOD), adrenocorticotropin (ACTH), gonadotropin-releasing hormone (GnRH), immunoglobulin-G (IgG), cortisol, heat shock protein-70 (Hsp-70), growth hormone (GH), insulin, lipid peroxide (LPO), and tumor necrosis factor-α (TNF-α) levels. The determination was carried out at room temperature according to the manufacturer’s instructions.

### 2.3. Statistical Analyses

A linear mixed model with repeated measurements was used to investigate the influence of season and sampling time on body weight, body height, oblique length, chest circumference, and plasma parameters of calves. The results were analyzed with SPSS software (IBM, Armonk, USA) using the model:

Y_ijkl_ = μ + C_i_ + S_j_ + T_k_ + (S × T)_jk_ + e_l_

where Y_i_ is the average value of body weight, body height, oblique length, chest circumference, or plasma parameters of calves; μ is the overall mean; C_i_ is the random effect of cow, S_j_ is the fixed effect of season (j = summer, winter); T_k_ is the fixed effect of time (k = number of day); (S × T)_jk_ represents the effect of the interaction between season and time; and e_i_ is a random residual.

## 3. Results

### 3.1. Environmental Data, Dam Physiological Parameters, and Qualified Rate of Colostrum

**Figure 1:**
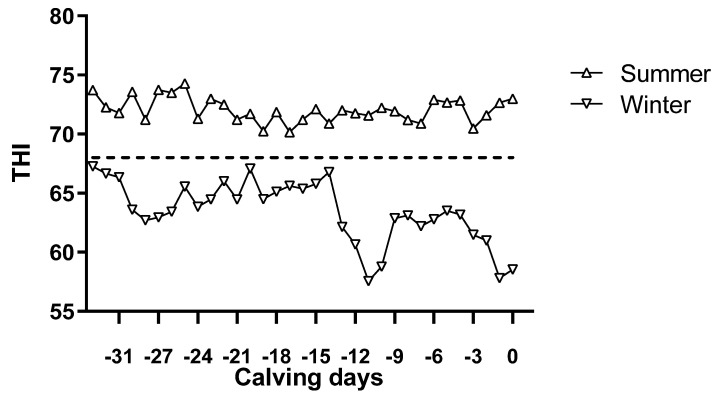
Late gestation average temperature–humidity index (THI) during summer and winter. The dashed line represents the THI threshold (68) when cows start to experience the effect of heat stress [25].

The patterns of THI values for both seasons are presented in Figure 1. The results showed that during the whole experiment period, the range of THI value was 70.15–74.28 in summer and 57.55–67.25 in winter. Based on the summer THI measurements, the late gestation cows were under heat stress. The daily average temperature of the late gestation cows in the winter was lower than that of the animals subjected to heat stress during the summer (*p* < 0.01, Table 2). The length of pregnancy in summer was shorter than in winter, but there was no significant difference (*p* = 0.33, Table 2). Compared with winter, the qualified rate of colostrum in summer decreased by 19.23% (Table 3).

### 3.2. The Growth Performances of Calves

**Figure 2:**
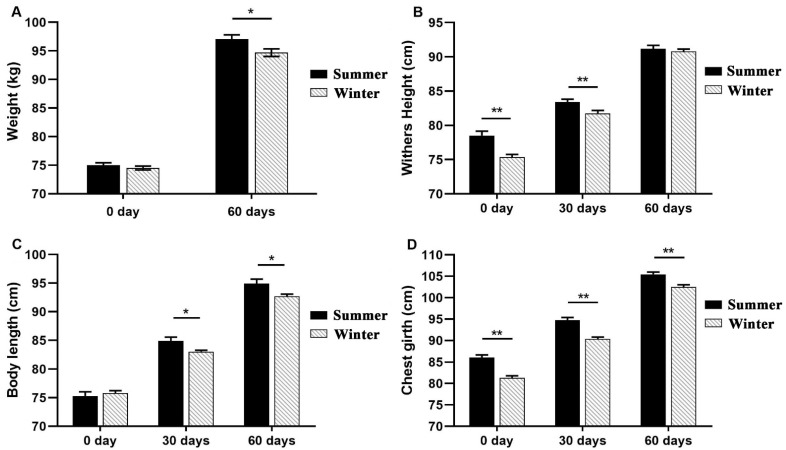
Effects of seasonal heat stress on growth performance of calves at birth (0) and at 30 or 60 days postpartum. (**A**) is the birth weight and weaning weight of the calf; (**B**) withers, (**C**) body length, (**D**) chest girth of calf at 0, 30 and 60 day. Error bars represent SEM. Significance is indicated at *p* < 0.05 (*) and *p* < 0.0l (**).

There was no significant difference in BW between seasons (*p* > 0.05), but calves from the summer group had a higher weaning weight (WW) (*p* < 0.05). There was no statistically significant difference in body length at birth between the summer group and the winter group (*p* > 0.05); however, there was a significant difference in WH and chest girth between the summer group and winter group at birth (*p* < 0.0l). In the summer group, calves’ WH, body length, and chest girth were significantly higher than those in the winter group (*p* < 0.05) at 30 days of age. There was no significant difference in weaning WH between the summer group and winter group. However, calves in the summer group had higher weaning body length and chest girth (*p* < 0.05) compared with calves in the winter group (Figure 2). Compared with the winter group, the one-week postpartum diarrhea rate of calves in the summer group increased by 35.40% (Table 3).

### 3.3. The Hormonal, Oxidative Stress, and Immune Parameters of Calves

**Figure 3:**
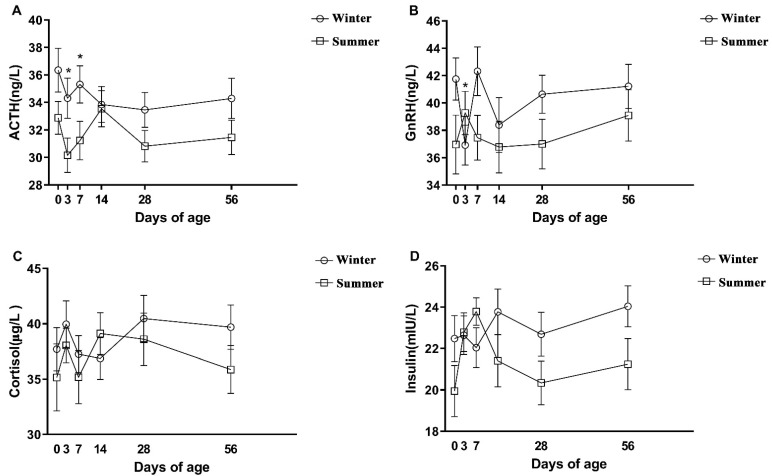
Seasonal heat stress in late gestation changes in plasma hormone concentrations at different ages in calves. Including (**A**) ACTH concentration of plasma, (**B**) GnRH concentration of plasma, (**C**) cortisol concentration of plasma, (**D**) insulin concentration of plasma. Error bars represent SEM. Significance is indicated at *p* < 0.05 (*).

The seasonal heat stress during late gestation significantly decreased the concentrations of ACTH, GnRH, and insulin in calves (*p* < 0.05, Table 4), and the concentrations of cortisol in calves showed no difference between the summer and winter groups (*p* = 0.19, Table 4). The concentrations of ACTH in the plasma of the summer group were significantly lower than those of the winter group at 3 and 7 days and declined at first and then increased at 0–14 d and 14–56 d, respectively (Figure 3A). The summer group’s GnRH concentrations fluctuated dramatically from 0 to 7 days, rising at first and then decreasing, and the summer group GnRH concentrations were considerably greater than the winter group at 3 days of age (*p* < 0.05, Figure 3B). The concentrations of insulin in the summer group increased rapidly in 0–7 d (Figure 3D), and the cortisol concentrations in the two groups changed sharply from 0 to 7 d, showing a trend of rising at first and then decreasing (Figure 3C).

**Figure 4:**
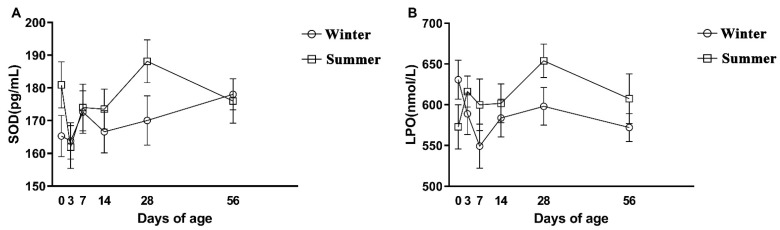
Late gestation changes in oxidation. Including (**A**) SOD concentration of plasma, (**B**) LPO concentration of plasma. Error bars represent SEM.

Compared to the winter group, the concentrations of SOD and LPO in the summer group were higher, but there was no significant difference (*p* > 0.05, Table 4). The two groups fluctuated greatly at different time points (Figure 4); SOD and LPO reached the maximum at day 28 then gradually decreased.

**Figure 5:**
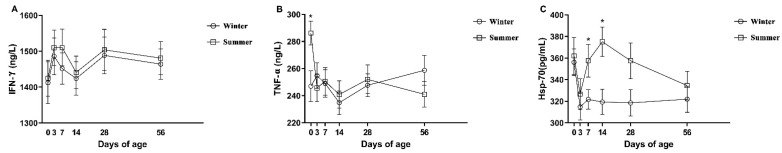
Seasonal heat stress in late gestation changes in plasma immune indexes at different ages in calves. Including (**A**) IFN-γ concentration of plasma, (**B**) TNF-α concentration of plasma, (**C**) hsp-70 concentration of plasma. Error bars represent SEM. Significance is indicated at *p* < 0.05 (*).

The results of immune indexes of calves showed that seasonal heat stress during late gestation increased the concentrations of IFN-γ, IgG, and TNF-α but these increases did not reach a significant level (*p* > 0.05, Table 4), meanwhile the level of HSP-70 was significantly increased (*p* < 0.05, Table 4). The concentrations of IFN-γ in the plasma of both groups increased at first and then decreased from 14 to 56 days (Figure 5A). The concentrations of TNF-α in the summer group were significantly higher than in the winter group at 0 d (*p* < 0.05, Figure 5B). From 7 to 28 d, the concentrations of TNF-α in both groups decreased at first and then increased (Figure 5B). In the summer group, plasma levels of Hsp70 decreased sharply in 0–3 d, increased rapidly after 3 days, then began to decrease after 14 days, while in the winter group, plasma levels of Hsp70 increased slowly and maintained a stable level after 3 days (Figure 5C). The concentrations of plasma Hsp70 were significantly higher in the summer group than in the winter group at days 7 and 14 (*p* < 0.05, Figure 5C).

## 4. Discussion

The unfavorable influence of heat stress will worsen with global warming. It can be seen that heat stress has a serious impact on animal production performance. Our study results showed that the rectal temperature of dry cows increased under heat stress, and these results were consistent with Fabris’s study [16]. Dairy cows experience reduced metabolic rates, vasodilation, and behavioural changes in response to heat stress, resulting in changes in blood hormone concentrations and body temperature [26]. Heat stress can also shorten the pregnancy time in dry dairy cows [22]. When a cow is subjected to prolonged heat stress, it utilizes a heat-regulating system to lower its body temperature, which increases energy consumption. These physiological responses will reduce the production performance of the dam and affect the calves. Heat stress in late gestation reduces the birth weight of newborn animals, which reflects impaired fetal development in utero. The causes of impaired fetal growth are reduced pregnancy [27] and malnutrition [28,29], but the determinants are placental development and function [2]. Circulating placental hormones and total uterine and umbilical blood flow decrease [30], and compromised placental vascularization is also observed in heat-stressed animals [31], which reflects the impairment of placental development. Reductions in total DNA, RNA, and protein contents in the placentas of heat-stressed animals have also been observed [32].

There has been a lot of research into the effects of heat stress on calves’ birth weights and weaning weights. Tao et al. [23] found that heat stress during late gestation significantly reduced the birth weight and weaning weight of calves, and active cooling treatment could counteract the effect of heat stress on calf birth weight. Unexpectedly, in our experiment, there was no significant difference in birth weight between the two groups. In the current experiment, similar gestation lengths in Summer and Winter dams provided fetuses from both groups with similar lengths of time in which to grow. In addition, similar calf birth weights shows that the extent of changes in the uterine thermal environment due to maternal heat stress are not sufficient to reduce fetal growth.

Insulin is essential for glucose and fatty acid metabolism, and its secretion and response changes in sheep fetuses and lambs due to heat stress during pregnancy [33,34,35]. Heat stress in the second trimester of pregnancy resulted in a greater insulin response to glucose in the lambs. Previous studies have shown that heat stress has no significant effect on calves’ insulin levels [14], while in our study heat stress reduced the plasma levels of insulin. It is possible that insulin secretion in newborns was significantly reduced due to the lack of nutrients during intrauterine development [36,37]. The lack of nutrients can be caused by heat stress [38]. From a physiological point of view, heat stress can activate the hypothalamic–pituitary–adrenal axis. The hypothalamus secretes corticotropin-releasing hormone (CRH), which stimulates the pituitary gland to secrete ACTH. However, in our experiment, we found an interesting phenomenon in which the plasma ACTH concentrations of calves decreased at first and then increased one week after birth. At 3 and 7 days of age, seasonal heat stress during late gestation significantly decreased the content of ACTH in the plasma of calves. We hypothesized that seasonal heat stress during late gestation affects the intrauterine development of fetal adrenal glands, thereby reducing ACTH secretion in newborn calves. Previous studies have also found that, under normal circumstances, space limitation in the uterus induces fetal stress during late gestation, leading to the release of adrenocorticotropic hormone or ACTH in the fetus [39]. Further research is needed to validate this supposition.

The balance between oxidants and antioxidants in newborn calves is impaired due to heat stress. Rannade et al. [40] found that calves showed respiratory oxidative stress during weaning. This study found that the plasma concentrations of SOD and LPO in calves increased before weaning. When the body is in a pathological condition, the lipid peroxidation reaction is enhanced, leading to an increase of LPO. SOD, as the primary member of the intracellular antioxidant defense, is considered an indicator of oxidative stress [41]. High levels of oxidative stress markers in serum indicate a general increase in oxidative stress in most tissues or increased oxidative stress in specific tissues [42]. Therefore, exposure to heat stress during late gestation will affect the oxides of calves.

The mechanism of the effect of uterine heat stress on postpartum liver function in late gestation has not been clarified, which may lead to poor performance of calves under intrauterine heat stress [10]. Monteiro et al. [14] found that calves born from cows subjected to heat stress during the dry period had a lower IgG absorption efficiency but no significant effect on serum IgG concentrations. In this experiment, there were no significant differences in IgG concentrations in plasma. In addition, in this experiment, it was observed that plasma IFN-γ decreased while TNF-α increased in the summer group, which suggests the possibility of an inflammatory condition resulting from the heat stress environment. This result was supported by the fact that oxidative stress caused inflammation [43], and plasma TNF-α and IL-6 were shown to increase under long-term heat stress in dairy cows [44]. IFN-γ has antiviral, immunomodulatory, and antitumor properties. IFN stimulates innate and adaptive immune mechanisms that help clear viral infections [45]. TNF-α is a multi-effect pro-inflammatory medium related to inflammation, infection, autoimmunity, and malignant diseases. When the body is infected, TNF is produced, providing immunity to the host. Although the role of TNF in infection is beneficial, it is necessary to strictly regulate the production of TNF to protect the host from the harmful activity of TNF. Overexpression of TNF can lead to chronic inflammation and autoimmune diseases, such as chronic inflammatory arthritis, inflammatory bowel disease (IBD), and multiple sclerosis [46]. TNF is the initiating factor of intestinal inflammation when TNF/TNFR1 genetic inactivation or the use of anti-TNF antibody and other methods can reduce or improve inflammation [47,48,49,50]. In our experiment, seasonal heat stress during late gestation led to a higher risk of diarrhea in calves. Heat shock proteins consist of highly preserved stress proteins, which are expressed during stress. Hsp can be classified as Hsp110, Hsp100, Hsp90, Hsp70, Hsp60, Hsp40, Hsp10, and small Hsp families [51]. Hsp 70 and Hsp90 were correlated with the development of heat resistance [52]. In farm animals, heat stress was shown to increase both Hsp70 and Hsp90 in buffalo [53] and cattle [54]. Our experiment also observed that seasonal heat stress during late gestation led to an increase in Hsp70 in calves. Although Hsps prevent their interaction with neighboring proteins, thereby preventing the loss of protein function, the increased Hsp70 and Hsp90 levels during heat stress may be due to an increase in the number of damaged proteins, as Hsps are induced as a mechanism for cellular defense and/or repair [55].

## 5. Conclusions

Despite a few limitations of our study, including the lack of data on BCS, respiration rates, and panting scores of cows, we identified that seasonal heat stress during late gestation can lead to effects on metabolism, oxidative stress, and immunity in calves. Understanding the impact of heat stress on calf development and metabolic and endocrine characteristics in late-gestation cows is important for preventing the negative consequences of heat stress on dairy cow production.

## Figures and Tables

**Table 1:** Test diet composition and nutritional level (%, dry matter basis).

Diets Composition and Nutritional Level	Dry Cows in Summer	Dry Cows in Winter
Diets Composition		
Corn silage	28.77	30.44
Oat grass	34.44	34.44
Perinatal concentrated feeding stuff	34.47	33.09
Sugarcane molasses	2.32	2.03
Nutritional Level		
DM	37.45	41.79
CP	22.75	23.84
NDF	38.42	40.04
ADF	23.77	24.94
NEL(MJ/kg)	1.42	1.55

**Table 2:** Rectal temperatures and gestation length from dairy cows during summer (*n* = 28) or winter (*n* = 23) during late gestation.

Variable	Summer	Winter	SEM	*p*-Value
Rectal temperature (°C)	39.02	38.95	0.02	<0.01
Gestation length (d)	274.00	275.44	1.51	0.33

**Table 3:** Qualified rate of colostrum and diarrhea rate of calves during one week after delivery.

Variable	Summer	Winter
Colostrum qualified rate (%)	80.77	100
Diarrhea rate of calves (%)	57.14	21.74

Colostrum qualified the Brix value ≥ 20% is defined as qualified colostrum.

**Table 4:** Plasma metabolism and endocrine concentrations of calves before weaning in summer and winter.

Variable	Summer	Winter	SEM	*p*-Value
IFN-γ (ng·L^−1^)	1480.93	1454.42	28.36	0.633
SOD (pg·mL^−1^)	175.24	169.42	3.77	0.058
ACTH (ng·L^−1^)	31.61	34.60	0.77	<0.001
GnRH (ng·L^−1^)	37.83	40.21	1.00	0.034
Cortisol (μg·L^−1^)	37.08	38.66	1.19	0.142
HSP-70 (pg·mL^−1^)	351.26	325.43	7.81	0.002
GH (μg·L^−1^)	23.56	23.92	0.57	0.625
Insulin (mIU·L^−1^)	21.67	22.95	0.61	0.038
LPO (nmol·L^−1^)	609.88	587.13	14.22	0.204
TNF-α (ng·L^−1^)	251.62	248.69	5.83	0.369
IgG (μg·mL^−1^)	2828.32	2747.20	66.75	0.829
